# Comparative Performance of Mechanistic, Statistical, and Hybrid Models of Forecasting Dengue Fever Incidence in Somalia. A Retrospective Time Series Analysis

**DOI:** 10.1002/hsr2.72822

**Published:** 2026-07-25

**Authors:** Abdiftah Mohamud Abdi, Saralees Nadarajah, Abdisalam Hassan Muse

**Affiliations:** ^1^ School of Postgraduate Studies and Research Amoud University, Amoud Valley Borama Somalia; ^2^ Department of Mathematics University of Manchester Manchester UK; ^3^ Research and Innovation Center Amoud University Borama Somalia

**Keywords:** dengue fever, forecasting, hybrid models, SIR model and Somalia, time series analysis

## Abstract

**Background and Aims:**

Dengue fever is a growing menace in Somalia, a climate change prone region with a weak healthcare system. An imperative of public health is effective forecasting models. This paper will provide a detailed comparative analysis comparing mechanistic, statistical, and hybrid models in order to find the best forecasting model to use in this data‐sparse situation of dengue fever.

**Methods:**

We utilized raw reported annual incidence data of dengue (1990–2021, *N* = 32) obtained from Our World in Data. Due to the lack of standardized case definitions and high under‐reporting inherent to Somalia's surveillance system, forecasts represent the projected reported burden rather than true infection rates. The data was divided into a training and a testing set (1990–2016 and 2017–2021, respectively). We tested a mechanistic Susceptible‐Infected‐Recovered (SIR) model, 8 single time series models (ARIMA, ETS, TBATS, and NNAR), and 12 hybrids using an averaging ensemble strategy.

**Findings:**

Although the time series models (individually) offered the basics of predictive power, the hybrid ARIMA‐TBATS model was the most successful and it surpassed the other models. It performed the highest accuracy statistics on the testing data with a Mean Absolute Percentage Error (MAPE) of 6.49% and a Root Mean Squared Error (RMSE) of 663.81 and outperformed the best single model (TBATS) which had a MAPE of 7.14%. The basic reproduction number ($R_{0}$) calculated by the SIR model was 1.015 which shows that the disease is endemic.

**Conclusion:**

This paper concludes that the ARIMA‐TBATS hybrid should be the most empirically effective tool out of the ones considered when planning to forecast dengue in Somalia. Real‐World Application: This ARIMA‐TBATS framework is designed to serve as the mathematical engine for a National Dengue Early Warning System (DEWS) in Somalia, allowing public health officials to physically preempt outbreaks by routing medical supplies, mobilizing fumigation teams, and distributing bed nets to high‐risk zones months before peak incidence occurs.

## 1 Introduction

The forecasting of infectious diseases is vital for public health and relies on two main approaches. Mechanistic models, like the SIR framework, explain the “why“ of an outbreak by simulating the actual transmission process. In contrast, phenomenological or statistical models are data‐driven, focusing on the “what” and “when” by identifying and extrapolating temporal patterns from historical data. The selection between these methods was ultimately determined by the specific research goals and the availability of reliable epidemiological data [1, 2, 3].

The application of models to forecast infectious diseases has a long history, initially focusing on well‐documented diseases before expanding to vector‐borne illnesses like dengue fever globally. Earnest et al. [4] compared autoregressive integrated moving average (ARIMA) and Bayesian Knorr‐Held models for dengue prediction in Singapore, while Akter et al. [5] found neural networks more effective for dengue forecasting in Dhaka, Bangladesh. Despite modeling efforts for diseases like malaria and Ebola in Africa, there is a noticeable lack of focus on dengue fever in the Horn of Africa [4, 5, 6]. This gap highlights the need for region‐specific models due to unique local factors.

Somalia presents a uniquely challenging context for dengue fever modeling due to its high susceptibility to climate change, which can expand the habitat of the mosquito vector. The nation grapples with a fragile health system, weakened by conflict, leading to limited public health infrastructure and weak disease surveillance. Consequently, reliable epidemiological data is often scarce, posing a significant obstacle to public health planning. Developing robust forecasting models is a public health imperative to create an early warning system. Such a system would enable the mobilization of resources and implementation of targeted vector control measures, ultimately saving lives [7, 8, 9].

Recent advancements in computational fluid dynamics increasingly rely on sophisticated numerical and stochastic frameworks to manage complex transmission dynamics and random fluctuations in micro‐ and nanoscale environments. Recent studies have demonstrated the efficacy of high‐order numerical schemes and two‐stage predictor‐corrector methodologies in addressing time‐dependent stochastic partial differential equations (SPDEs). Specifically, Arif et al. (2025a) and Arif et al. (2025b) have highlighted the robustness of second‐order stochastic computational schemes in capturing the interplay between electro‐osmotic effects and energy dissipation. Furthermore, investigations into fractal stochastic models (Arif et al., 2024) and their application in mixed convective nanofluid flows have provided novel insights into faster convergence rates compared to traditional techniques. These state‐of‐the‐art approaches underscore the growing necessity of integrating random perturbations and compact discretization methods to ensure both numerical stability and physical realism in stochastic simulations [10, 11, 12].

The mechanistic SIR model is a cornerstone of epidemiology, offering significant insights into disease dynamics by modeling population shifts between susceptible, infected, and recovered states. This model is instrumental in estimating the basic reproduction number $R_{0}$ and evaluating the effectiveness of interventions such as vaccination and social distancing. However, its practical application was severely limited by its dependence on precise parameters like transmission and recovery rates. In regions with inconsistent data collection, such as Somalia, obtaining accurate parameters is a major challenge, thus compromising the model's real‐time forecasting capabilities and highlighting the need for alternative methods [13].

Time series models offer a powerful way to forecast disease by using historical data to identify future trends. A comprehensive suite of models, including ARIMA, error, trend, seasonality (ETS), TBATS, and neural networks, was considered, each with unique strengths. These models can capture linear relationships, handle trend and seasonality, or model complex non‐linear patterns inherent in epidemic data. The core objective is to evaluate these diverse frameworks to find the most robust model specifically for Somali dengue data [14, 15].

Hybrid forecasting represents a state‐of‐the‐art approach, addressing the complexity of disease transmission by combining different models. This technique decomposes a time series into simpler parts, using the most suitable model for each component to enhance predictive power. A compelling local precedent in Somalia showed an ARIMA‐TBATS hybrid model outperformed individual models in forecasting CO_2_ emissions. Applying this successful hybridization philosophy to dengue forecasting is therefore a logical and innovative next step for the region [16].

### 1.1 Motivation Statement

This research was driven by the urgent need to combat the growing threat of dengue fever in Somalia, a country with a fragile healthcare system and scarce data, which hinders effective outbreak response. The core problem is the absence of a validated and reliable forecasting model specifically adapted to Somalia's challenging data environment, which was marked by under‐reporting and gaps. To address this, the study will systematically compare a range of forecasting models from traditional epidemiological models to advanced hybrid approaches to identify the most accurate and practical method for the local context. The ultimate goal is to develop a quantitative foundation for a dengue early warning system, empowering Somali health authorities and their partners to proactively allocate resources, implement timely interventions, and save lives in a highly vulnerable setting.

### 1.2 Novelty of the Paper

The novelty of this paper lies in its three‐pronged approach within a critically under‐researched geographical context. It was the first study to conduct a comprehensive, side‐by‐side comparative analysis of mechanistic (SIR), a wide array of single time series, and advanced hybrid models for forecasting dengue fever specifically in Somalia. While individual modeling techniques have been applied to dengue elsewhere, this research moves beyond a one‐model‐fits‐all approach to perform a rigorous, evidence‐based evaluation to identify the optimal forecasting framework for a data‐scarce, high‐vulnerability setting. The direct comparison of these methodologically distinct classes of models, particularly the inclusion of sophisticated hybrid structures like ARIMA‐TBATS, in such a challenging data environment represents a significant and novel contribution to the field of infectious disease forecasting.

### 1.3 Rationale Behind the Selection of the Models

The selection of models for this study was deliberately systematic, aiming to compare a broad spectrum of forecasting methods. A mechanistic susceptible‐infected‐recovered (SIR) model was chosen to serve as a theory‐driven baseline, useful for understanding epidemic dynamics, particularly in data‐poor contexts. Complementing this, a diverse set of eight single time series models, including ARIMA, ETS, and neural network approaches like neural network autoregressive (NNAR) and MLP, were selected to capture a variety of temporal patterns, from linear trends and autocorrelation to complex seasonalities and non‐linear relationships. Furthermore, state‐of‐the‐art hybrid models were included, which were predicated on the principle that combining the strengths of different models such as an ARIMA for linear components and a neural network for non‐linear residuals can overcome individual limitations and produce more accurate forecasts. The inclusion of these hybrid models is crucial for determining if a synergistic approach can provide the most accurate forecasts for dengue in Somalia.

### 1.4 Main Objective of the Paper

The key aim of this paper is to obtain a systematic comparison of a total of 21 varying modeling frameworks, including 1 mechanistic model of SIR, 8 single time series models (ARIMA, ETS, THETA, BATS, TBATS, NNAR, autoregressive fractionally integrated moving average (ARFIMA), and MLP), and 12 of their hybrid combinations in order to predict the occurrence of dengue fever in Somalia. The results of these models will be critically compared with the help of a set of forecasting measures determining which of the forecasting models will be considered the most accurate, the most reliable, and practically efficient forecasting model on the data conditions of Somalia.

### 1.5 What This Paper Will Achieve

This paper will achieve several critical outcomes. First, it will provide the first scientifically validated, optimal forecasting tool for dengue fever in Somalia, offering a crucial piece of evidence‐based infrastructure for the country's public health system. Second, it will generate short‐to‐medium‐term forecasts of dengue incidence, providing actionable intelligence for immediate planning and resource allocation. Third, by systematically comparing a wide range of models, it will offer a methodological roadmap for other researchers and public health practitioners working on disease forecasting in similar data‐scarce and resource‐constrained environments. Finally, the findings will contribute to the broader academic literature on the comparative performance of different forecasting paradigms in real world, high‐stakes public health scenarios.

### 1.6 Outline of the Paper

The remainder of this paper was structured as follows. The Materials and Methods section detail the study design, data sources and preprocessing steps, the methodology for stationarity testing, the software used, and a comprehensive description of the development and evaluation of the mechanistic, single time series, and hybrid models. The Results section presents the findings of the comparative analysis, including model performance metrics and the forecasts generated from the optimal model. The Discussion section interprets these findings in the context of existing literature and the practical realities of public health in Somalia. Finally, the Conclusion summarizes the key contributions of the study and provides actionable recommendations for public health stakeholders and future research.

## 2 Materials and Methods

### 2.1 Study Design and Data Source

This study employs a retrospective time series analysis design. Historical data on the annual incidence of dengue fever in Somalia were sourced from “Our World in Data,” which aggregates data from various public health organizations (Ritchie et al., 2024). The dataset spans the longest available period to provide a sufficient number of observations for model training and validation. While 32 annual observations represent a limited sample size that inevitably masks critical intra‐seasonal dynamics (such as post‐monsoon transmission spikes), it constitutes the longest and most comprehensive epidemiological record available for this conflict affected region. Consequently, this annual analysis serves as a necessary macro‐level baseline for early warning systems where high‐resolution temporal data does not currently exist.

### 2.2 Data Preprocessing

Prior to analysis, the dataset was preprocessed to ensure its suitability for time series modeling. This involved checking for and addressing any missing values or inconsistencies. The annual incident data were then formatted into a time series object for analysis in the R statistical environment. Given the weak healthcare system, these figures likely represent a severe undercount of true infections. Because no empirically validated multiplier exists for Somalia to correct for under‐reporting, the raw data was used without adjustment. Consequently, our modeling forecasts the future reported burden on healthcare infrastructure rather than true biological prevalence.

### 2.3 Stationarity Testing (ADF and KPSS Tests)

Stationarity testing is a crucial step in time series analysis to determine if a dataset's statistical properties, such as mean and variance, are constant over time. The Augmented Dickey‐Fuller (ADF) test checks for a unit root, where a p‐value below 0.05 suggests the data is stationary. Conversely, the Kwiatkowski‐Phillips‐Schmidt‐Shin (KPSS) test assumes stationarity, and a p‐value above 0.05 indicates the data is non‐stationary. These tests are essential for selecting the appropriate forecasting models, as many require stationary data for accurate predictions.

Formula Stationarity Testing (ADF and KPSS tests)

(1) $$\mathrm{ADF}=\frac{\text{Coefficient Estimate}-1}{\text{Standard Error}}\;$$

### 2.4 Software and Research Process

All data analysis, model development, and forecasting were conducted using the R statistical software environment (Version 4.0 or later). The research process followed a structured sequence: (1) data preprocessing and visualization; (2) stationarity testing; (3) splitting the data into a training set (for model fitting) and a testing set (for out‐of‐sample evaluation); (4) systematic development and fitting of the SIR, 8 single time series, and 12 hybrid models on the training data(1990‐2016); note that the SIR model was calibrated differently, utilizing the stabilization period (2000–2020) to extract biologically plausible contemporary parameters; (5) evaluation of the forecasting accuracy of all models on the testing set using multiple error metrics; and (6) generation of final forecasts using the best‐performing model.

### 2.5 Time Series Model Concepts

#### 2.5.1 Autoregressive Integrated Moving Average

The ARIMA model is a prominent statistical technique for forecasting time series data. It incorporates an Autoregressive (AR) component that uses past values to predict future ones and a Moving Average (MA) part that models the error term. The Integrated (I) element involves differencing the data to make it stationary, a prerequisite for the model. The model's structure is defined by the orders (p, d, q), which can be automatically optimized using functions like auto. Arima. As established by Box and Jenkins, the formula for the ARIMA (p, d, q) model is [17].

(2) $$\left(1-\emptyset_{1}L-\emptyset_{2}L^{2}-\ldots -\emptyset_{P}L^{P}\right){\left(1-L\right)}^{d}y_{t}=\left(1+\theta_{1}L+\theta_{2}L^{2}+\ldots -\theta_{p}L^{q}\right)\varepsilon_{t}\;.$$

#### 2.5.2 Error, Trend, Seasonality Model

The ETS model is a comprehensive framework for time series forecasting based on exponential smoothing within a state‐space approach. It decomposes a time series into three fundamental components: Error (E), Trend (T), and Seasonality (S). The framework is highly flexible because each component could be specified as either additive, multiplicative, or absent, allowing it to adapt to a wide variety of time series patterns. For instance, the trend can be linear (additive) or exponential (multiplicative) and could be “damped” to converge to a constant level over time. This adaptability makes the ETS model particularly effective at capturing various forms of heterogeneity and non‐linearity in the data. The state space equations of the ETS model can be written as follows:

(3) $$y_{t}=w\;(x_{t}-1)+r\;(x_{t}-1)\;\varepsilon_{t}\text{And}\;x_{t}=f\;(x_{t}-1)+g\;(x_{t}-1)\;\varepsilon_{t}.$$

#### 2.5.3 Neural Network Autoregressive Model

The NNAR model is a forecasting tool that combines the principles of auto‐regression with the non‐linear capabilities of neural networks. It uses past (lagged) values of the time series as inputs to a neural network to learn complex patterns and relationships that linear models like ARIMA might miss. A typical NNAR model consists of an input layer, one or more hidden layers, and an output layer connected by acyclic connections. For seasonal data, the model can be specified as NNAR (p, P, K) m, where it uses ‘p’ non‐seasonal lags and ‘P’ seasonal lags as inputs, processed through ‘K’ nodes in the hidden layer, for a time series with a seasonal period of ‘m’. This approach allows for more accurate forecasting when the underlying data‐generating process is non‐linear.

(4) $$y_{t}=\omega_{0}+\sum_{j=1}^{9}\omega_{j}g(\;\omega_{0j}+\sum_{i=1}^{p}\omega_{i,j}y_{t-1})+\varepsilon_{t}.$$

#### 2.5.4 TBATS Model

The TBATS model, which stands for Trigonometric seasonality, Box‐Cox transform, ARMA errors, Trend, and Seasonality, is a sophisticated and versatile time series model designed to handle complex seasonal patterns. It is particularly adept at modeling time series with multiple seasonal cycles (e.g., daily, weekly, and yearly patterns). The model uses trigonometric functions within a Fourier series representation to capture these intricate seasonalities. It also incorporates a Box‐Cox transformation to stabilize variance, an exponential smoothing state‐space model for the level and trend, and an ARMA process to model the residuals, making it a robust tool for challenging forecasting tasks.

(5) $${y_{t}}^{\left(\omega \right)}=l_{t-1}+\phi b_{t-1}+\sum_{t=1}^{T}{s^{\left(i\right)}}_{t-m_{i}}+d_{t}$$

### 2.6 Autoregressive Fractionally Integrated Moving Average

The ARFIMA model is an extension of the standard ARIMA model designed specifically for time series that exhibit long‐term dependencies, also known as long memory. While the ‘d’ parameter in ARIMA was an integer representing the number of times the series was differenced, ARFIMA allows 'd’ to be a fractional value. This fractional differencing provides a more flexible way to model the slow decay of correlations over long lags, which is characteristic of long‐memory processes found in fields like finance and hydrology. The ARFIMA (p, d, q) model formula was

(6) $$\left(1-\emptyset_{1}L-\emptyset_{1}L^{2}-\ldots -\emptyset_{P}L^{P}\right){\left(1-\theta_{1}L-\theta_{1}L^{2}-\ldots -\theta_{p}L^{q}\right)y}_{t}=\varepsilon_{t}.$$

### 2.7 Theta Model

The Theta model is a simple yet remarkably effective forecasting method, often described as a “random walk with drift” model. It was based on the concept of decomposing the time series and extrapolating its components. The core idea is that the future value of a time series could be represented as a linear function of its last observed value, augmented by a drift term that captures the long‐term trend. Despite its simplicity, the Theta model has proven to be a strong benchmark and often performs as well as more complex models in forecasting competitions. Following Hyndman and Billah [18], the formula of the theta model is as follows [18].

(7) $$y_{t+1}=\left(1+\theta \right)y_{t}+\mu +\varepsilon_{t}+1.$$

The system of ordinary differential equations (Equation 7) was solved numerically utilizing a 4th‐order Runge‐Kutta (RK4) algorithm within the R statistical environment (via the *deSolve* package), iterating over annual time steps to simulate compartmental flow.

### 2.8 Hybrid Modeling Approaches

Hybrid modeling is a forecasting strategy that combines the strengths of multiple different time series models to achieve greater accuracy. By integrating techniques like ARIMA, ETS, NNAR, and TBATS, these approaches can simultaneously capture various data characteristics, such as linear patterns (with ARIMA), non‐linear trends (with NNAR), and complex seasonality (with TBATS). To determine the optimal combination of models, we employed a simple, equal‐weight averaging ensemble strategy, where each constituent model's forecast contributes 50% to the final hybrid output. Equal weighting was deliberately chosen over inverse‐variance weighting to prevent severe overfitting on our small, highly volatile training dataset. The ARIMA‐TBATS combination was hypothesized to perform exceptionally well based on recent local precedents [16], as it theoretically combines ARIMA's proficiency in handling linear auto‐correlated trends with TBATS's ability to model complex, shifting variances. Finally, 11 other combinations were systematically included in our analysis to rule out alternative synergies, such as combining linear models with non‐linear neural networks (NNAR).

### 2.9 Mathematical Modeling of SIR Models

The SIR model categorizes individuals into three compartments: susceptible (S), infected (I), and recovered (R). The transitions between these compartments were governed by parameters that reflect the rate of transmission and recovery. This model relies on differential equations to describe the flow of individuals between compartments over time, making it an essential tool for understanding and predicting the spread of dengue fever in populations. While traditionally applied to short‐term, continuous‐time outbreaks, the SIR model with vital dynamics (births and deaths) was utilized in this study over a multi‐year span to establish a theoretical, macro‐level understanding of long‐term disease persistence and to calculate the baseline endemicity threshold (R_0_) of the region.

### 2.10 Derived Formula of SIR Model

(8) $$\frac{\mathrm{dS}}{\mathrm{dt}}=\mu N-\beta \frac{\mathrm{SI}}{N}-\mu S,$$

(9) $$\frac{\mathrm{dI}}{\mathrm{dt}}=\beta \frac{\mathrm{SI}}{N}-\left(\gamma +\mu \right)I,$$

(10) $$\frac{\mathrm{dR}}{\mathrm{dt}}=\gamma I-\mu R.$$

o μN: Represents the birth rate (all newborns enter the S compartment).
o −μS,−μI,−μR: Represents the death rate (natural mortality) in each compartment.
o −(γ+μ)I: Reflects that an infected individual leaves the I compartment either by recovery (γ) or death (μ).

#### 2.10.1 Parameter Estimation and Model Initialization

Parameter estimation in SIR models involves determining the values of β and γ that best fit the observed data. This could be achieved using various methods such as maximum likelihood estimation (MLE) or least squares optimization. The initial conditions must also be specified, typically the initial values of S, R., and I For instance, one might start with the initial number of susceptible individuals equal to the total population minus the number of initially infected individuals.

#### 2.10.2 Basic Reproduction Number and Stability Analysis

The basic reproduction number $R_{0}$ is a critical parameter that indicates the average number of secondary infections produced by a single infected individual in a wholly susceptible population. For the SIR model, it was given by:

(11) $$R_{0}=\frac{\beta }{\gamma +\mu }$$

Stability analysis involves examining the equilibrium points of the model and determining their stability, usually through linearization of the system of equations around these points. This is the “next‐generation matrix” result for an SIR model with demography. $R_{0}$ is the transmission rate (β) divided by the total rate at which an individual leaves the infected class (recovery + mortality, γ+μ).

#### 2.10.3 SIR Model Jacobian and Its Formula

The Jacobian matrix for the SIR model, evaluated at an equilibrium point (S*, I*, R*) was given by:

(12) $$J=\left[\begin{array}{lll} -\frac{\beta I}{N}-\mu & -\frac{\beta S}{N} & 0\\ \frac{\beta I}{N} & \frac{\beta I}{N}-(\gamma +\mu ) & 0\\ 0 & \gamma & -\mu \end{array}\right]$$

The term −μ is now present on the diagonal elements (corresponding to the derivatives with respect to S,I, and R), as the death rate μ acts as a constant removal factor across all three population compartments. This ensures the matrix correctly reflects the stability of the system with vital dynamics.

### 2.11 Model Evaluation

Model evaluation is a crucial step in assessing time series model performance by dividing data into training and validation sets. A model's predictive accuracy was then measured by comparing its forecasts against the actual values in the validation set using metrics like root mean squared error (RMSE), MAE, and mean absolute percentage error (MAPE). The model exhibiting the lowest error metrics was deemed the most accurate and chosen for forecasting. This process is vital for making informed predictions and developing effective policies in areas such as public health and population studies.

#### 2.11.1 Measuring Forecast Accuracy

Mean Absolute Error (MAE): Mean absolute error is a commonly used metric to quantify overall forecast error. According to Heifer and Render (2001), this value is calculated as follows: the sample size (the number of forecast periods) is divided by the sum of the absolute values of each individual forecast error [19] The equation is:

(13) $$\mathrm{MAE}=\frac{1}{n}\sum_{n=1}^{n}\;\left|\mathrm{actual}-\mathrm{forecast}\right|\;$$

### 2.12 Mean Absolute Percentage Error

The most commonly used indicator of forecast error is the MAPE. When the data has no extreme values, including zero, MAPE performs best. If the value is zero or almost zero, MAPE may provide an inaccurate error picture. If the error was averaged for an element close to zero, the error can be infinitely high, distorting the overall error rate. Symmetrical Mean Absolute Percentage Error is a more appropriate metric for forecasting items whose volume is near or zero. The average absolute percent error for each forecast period or actual minus actual divided by actual was called the MAP [20].

(14) $$\mathrm{MAPE}=\frac{1}{n}\sum_{n=1}^{n}\frac{|\mathrm{actual}-\mathrm{forecast}|}{\mathrm{actual}}\;*\;100\%$$

### 2.13 Root Mean Squared ErrorRMSE

The square root of MSE is called RMSE. It calculates the standard deviation of the error mathematically. Like MSE, RMSE is often used in model estimation and regression that requires numerical predictions.

(15) $$\mathrm{RMSE}=\sqrt{\frac{1}{n}}\sum_{i=1}^{n}{\left(y_{i}-\hat{y_{i}}\right)}^{2}$$

### 2.14 Mean Error

The difference between the expected and actual values is called the forecast error, and the mean error (ME) is a measure of the average of these errors. It can provide insight into whether forecasts typically overestimate or underestimate actual values

(16) $$\text{Mean Error}\;(\text{ME})=\frac{1}{n}\sum_{n=1}^{n}\left(\mathrm{forecast}-\mathrm{actual}\right)$$

## 3 Results

### 3.1 Descriptive Statistics and Stationarity

**Figure 1:**
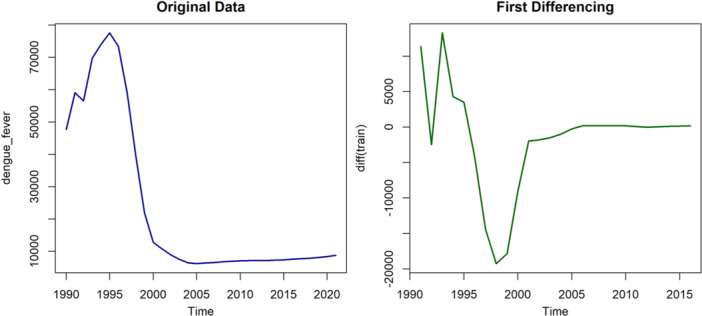
Data visualization.

Based on the 32 annual observations summarized in Table 1, the dengue incidence variable (X_1_) exhibits a highly dispersed and strongly positive skew. This is evident as high‐value outliers, compared to the much lower median of 7983, significantly inflate the mean to 23,412.16. The substantial standard deviation (25,453.96) and a wide range (71,383) confirm the data's considerable variability over the study period. As shown in Table 1, the positive skewness value of 1.08 indicates a long tail to the right, while the negative kurtosis (‐0.6) suggests a distribution that is flatter than normal with fewer extreme outliers. A visual representation of this volatility is provided in Figure 1 (left), which illustrates the dramatic peak in infections during the mid−1990s followed by a sharp decline and subsequent stabilization.

**Table 1:** Descriptive Statistics of the data.

Descriptive Statistics of the data	
Variables	$X_{1}$
Number of observations	32
Mean	23,412.16
Standard deviation	25,453.96
Median	7983
Trimmed Mean	19,427.92
Mean Absolute deviation	2014.11
Minimum	6188
Maximum	77,571
Range	71,383
Skewness	1.08
Kurtosis	−0.6
Square Error	4499.67

The results of the stationarity tests are presented in Table 2. The original dengue fever data was found to be non‐stationary; the ADF test yielded a *p*‐value of 0.05, failing to definitively confirm stationarity, while the KPSS test's *p*‐value of 0.01 strongly indicated the presence of a unit root. To address this, the data underwent “first differencing. As illustrated in Figure 1 (right), this transformation effectively stabilized the mean of the series by emphasizing year‐over‐year changes rather than absolute levels. Detailed in Table 2, after this transformation, the ADF test yielded a p‐value of 0.01 and the KPSS test showed a p‐value of 0.10, both of which confirm that the differenced data is stationary and suitable for rigorous time series modeling.

**Table 2:** Stationarity test results.

Stationarity test results		
Test	Test‐Statistics	*p* value
Augmented Dickey‐Fuller Test (Original data)	−3.5868	0.05
Augmented Dickey‐Fuller Test (first differencing)	−6.3477	0.01
Kwiatkowski‐Phillips‐Schmidt‐Shin Test (original data)	0.71421	0.01
Kwiatkowski‐Phillips‐Schmidt‐Shin Test (first differencing)	0.12348	0.10

### 3.2 Performance of Single Time Series Models

**Figure 2:**
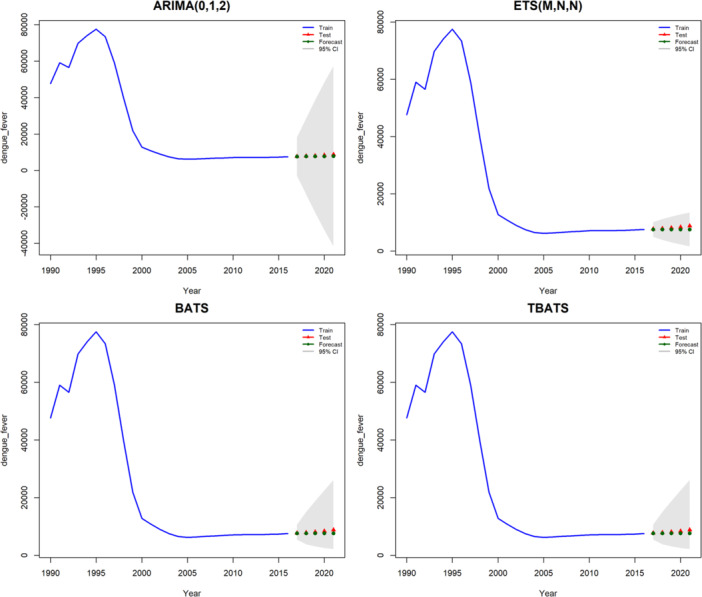
Single time series models forecasts (ARIMA, ETS, BATS, and TBATS Models).

An evaluation of individual time series models, detailed in Table 3, reveals that the TBATS and BATS models were the most effective single‐framework tools for forecasting dengue fever in Somalia. Both models demonstrated the lowest error rates across all metrics, specifically achieving a MAPE of 7.14% and a RMSE of 722.16. The superior fit of these models is visually confirmed in Figure 2, where the fitted lines (red) and forecasted points (green) closely track the actual historical trend (blue).

**Table 3:** Performance of single time series models.

Forecast performance of the single time series models					
Model	ME	RMSE	MAE	MPE	MAPE
ARIMA (0,1,2)	730.814	949.594	861.969	8.745	10.486
ETS	680.846	794.998	680.846	8.223	8.223
BATS	594.179	722.162	594.179	7.144	7.144
TBATS	594.179	722.162	594.179	7.144	7.144
THETA	5776.478	6459.561	5776.478	70.145	70.145
NNAR	949.973	1040.069	949.973	11.566	11.566
ARFIMA	−3052.474	3514.888	3052.474	−36.948	36.948

**Figure 3:**
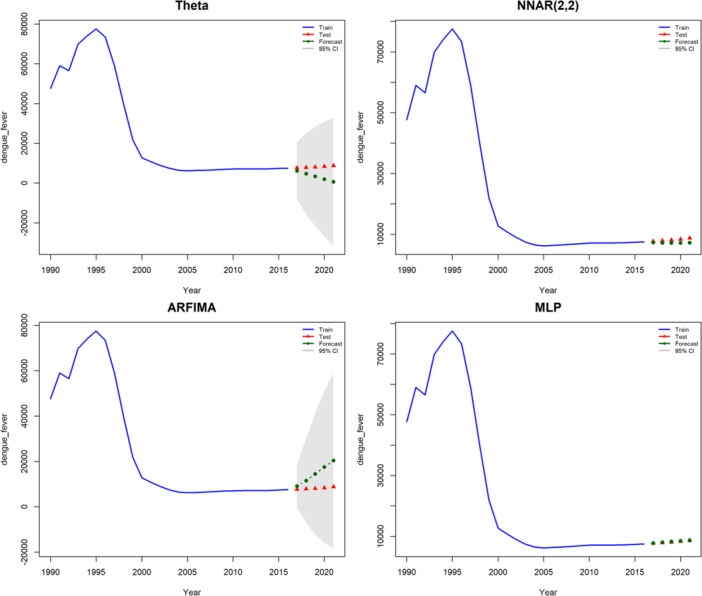
Single time series models forecasts (Theta, NNAR, ARFIMA, and MLP Models).

In contrast, as shown in Table 3, the THETA and ARFIMA models showed significantly poorer performance, with MAPEs of 70.15% and 36.95%, respectively, suggesting they are less suitable for capturing the complex dynamics of dengue transmission in this context. The failure of these models to capture the underlying dynamics is evident in Figure 3, where the forecasted values for Theta and ARFIMA deviate sharply from the actual test data points, suggesting they are less suitable for the complex epidemiological context of Somalia.

### 3.3 Performance of Hybrid Models and Comparative Analysis

**Figure 4:**
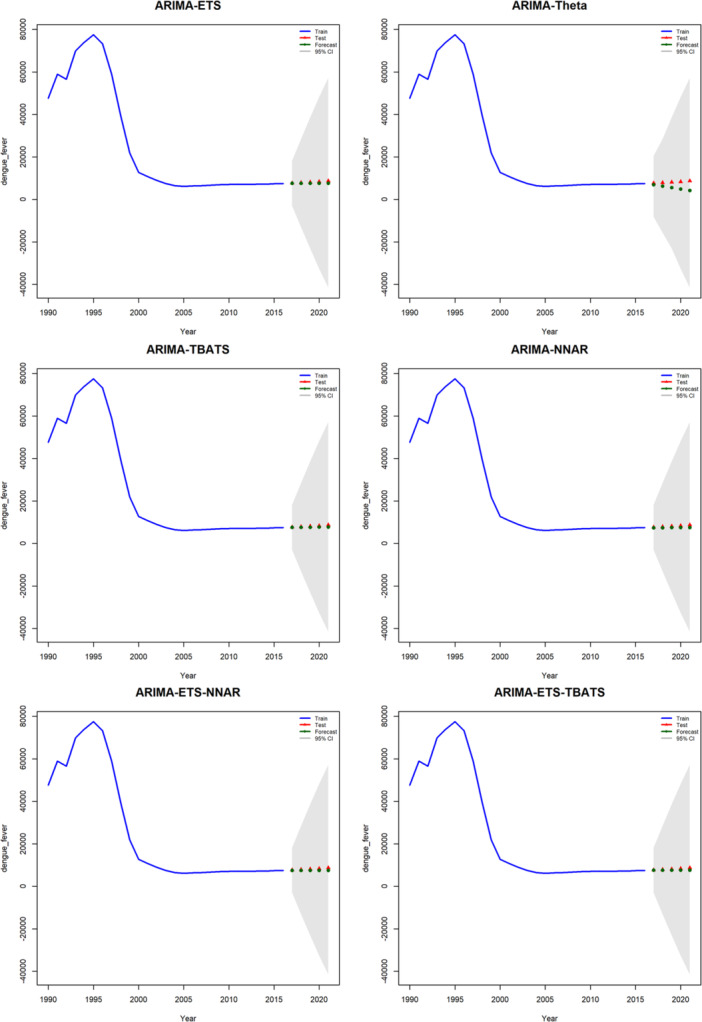
Hybrid models grid 1 forecasts.

**Figure 5:**
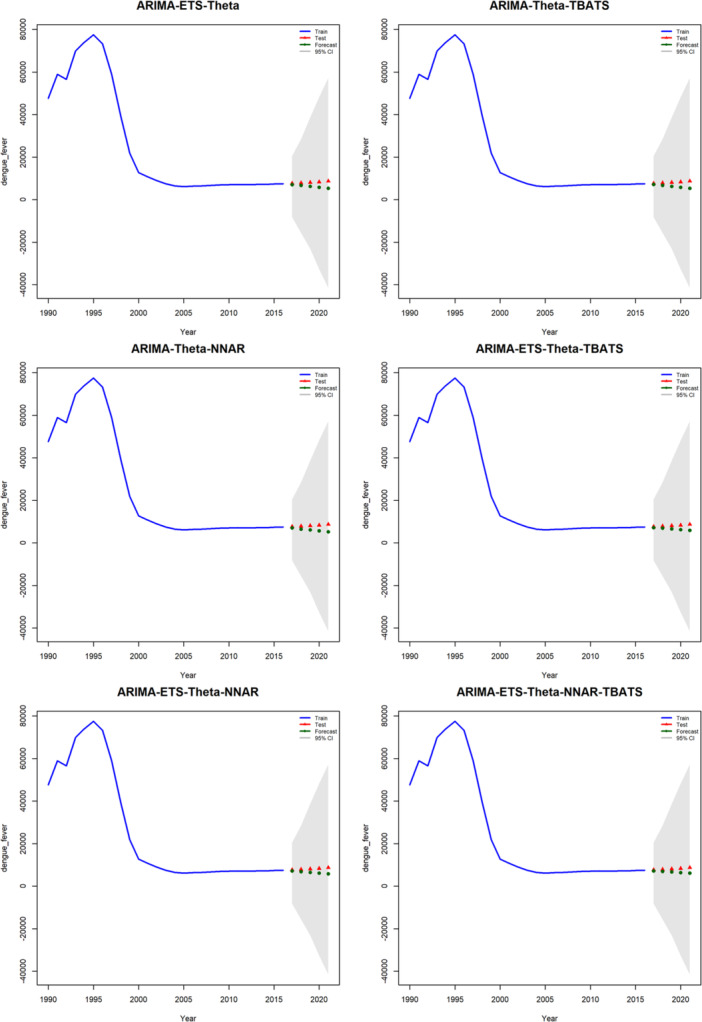
Hybrid models grid 2 forecasts.

Further analysis of the hybrid time series models, presented in Table 4, demonstrates the superior performance of the ARIMA‐TBATS combination. This hybrid model achieved the lowest MAPE of 6.49% and the lowest RMSE of 663.81, outperforming all other evaluated frameworks. Figure 4 and Figure 5 provide a side‐by‐side visual comparison of the 12 hybrid combinations. In these figures, the ARIMA‐TBATS model (Figure 4) demonstrates a unique ability to smooth out historical volatility while accurately capturing the stabilization trend observed in the test set (red triangles). Physically, the superiority of the ARIMA‐TBATS hybrid (Table 4) occurs because the raw aggregate data contains two distinct epidemiological realities: a slow‐moving, linear demographic trend (captured by ARIMA) and volatile, non‐linear environmental transmission spikes (captured by TBATS). Single models fail because they force a single mathematical paradigm onto dual physical dynamics.

**Table 4:** Performance of hybrid models.

Forecast performance of the hybrid time series models					
Model	ME	RMSE	MAE	MPE	MAPE
ARIMA‐ETS	584.16	699.57	584.16	7.03	7.03
ARIMA‐Theta	3131.98	3526.87	3131.98	37.99	37.99
ARIMA‐TBATS	540.83	663.81	540.83	6.49	6.49
ARIMA‐NNAR	640.91	750.66	640.91	7.73	7.73
ARIMA‐ETS‐NNAR	683.47	790.63	683.47	8.26	8.26
ARIMA‐ETS‐TBATS	683.47	790.63	683.47	8.26	8.26
ARIMA‐ETS‐Theta	2314.93	2615.26	2314.93	28.07	28.07
ARIMA‐Theta‐TBATS	2286.04	2589.72	2286.04	27.71	27.71
ARIMA‐Theta‐NNAR	2330.00	2629.24	2330.00	28.25	28.25
ARIMA‐ETS‐Theta‐TBATS	1884.74	2140.49	1884.74	22.84	22.84
ARIMA‐ETS‐Theta‐NNAR	1973.16	2220.27	1973.16	23.93	23.93
ARIMA‐ETS‐Theta‐NNAR‐TBATS	1672.53	1897.15	1672.53	20.27	20.27

The results in Table 4 underscore the value of the ensemble averaging strategy, as the hybrid approach effectively captures multifaceted patterns that individual models miss.

### 3.4 Forecast Uncertainty and Model Selection

**Figure 6:**
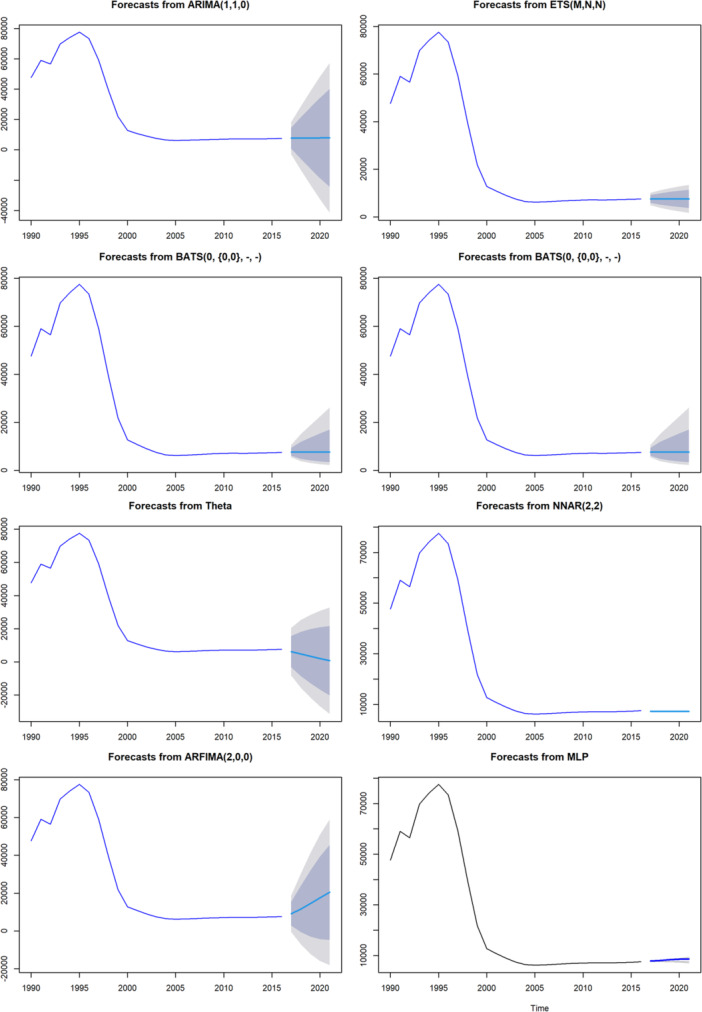
Forecast uncertainty for the single models.

**Figure 7:**
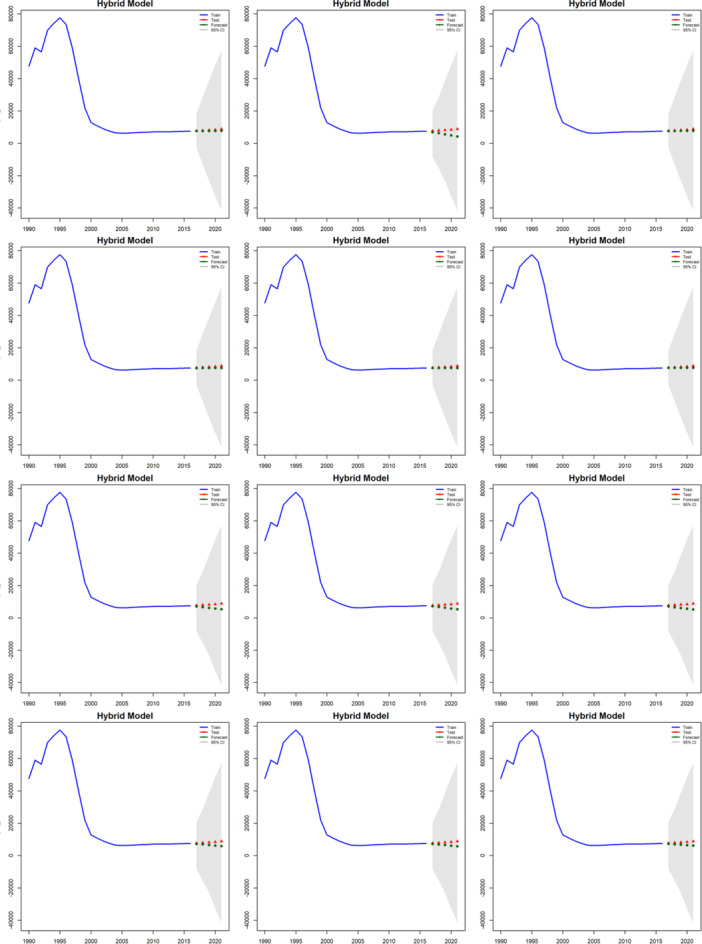
Comprehensive 12‐grid comparison of all hybrid models.

A critical aspect of the forecasting evaluation involved assessing prediction intervals. Figure 6 displays the forecast uncertainty for the single models, characterized by the progressive widening of the shaded confidence intervals. This visualization highlights the increasing uncertainty inherent in long‐term projections, a concern specifically noted during the model validation process. To search for the most stable framework, Figure 7 presents a comprehensive 12‐grid comparison of all hybrid models, allowing for a visual assessment of how different combinations handle the “widening” uncertainty.

A direct comparison between the best‐performing single model (TBATS) and the top hybrid model (ARIMA‐TBATS) is provided in Table 7. The ARIMA‐TBATS model registered lower values across every error metric, including a Mean Error (ME) of 540.83 compared to 594.18 for the TBATS model. This comparison, as summarized in Table 7, confirms that integrating the linear strengths of ARIMA with the seasonal flexibility of TBATS leads to a more robust and reliable forecasting tool. To ensure practical utility for decision‐makers, specific point forecasts alongside strict 95% upper and lower prediction intervals for the years 2022 to 2030 are provided in Table 5 (for TBATS) and Table 6 (for ARIMA‐TBATS), projecting a stabilization of incidence levels in the coming decade.

**Table 5:** Forecast values of number of dengue fever during 2022–2030 using the best fitted of single time, series models (TBATS).

Model	year	Point forecast	95% lower bound	95%‐upper bound
TBATS	2022	8991.208	6574.538	12296.20
TBATS	2023	8991.208	4772.440	16939.30
TBATS	2024	8991.208	3884.446	20811.67
TBATS	2025	8991.208	3295.232	24532.96
TBATS	2026	8991.208	2861.571	28250.84
TBATS	2027	8991.208	2523.994	32029.31
TBATS	2028	8991.208	2251.616	35903.90
TBATS	2029	8991.208	2026.235	39897.52
TBATS	2030	8991.208	1836.200	44026.67

**Table 6:** Forecast values of number of dengue fever during 2022 to 2030 using the best fitted of hybrid time, series models (ARIMA‐TBATS).

Model	year	Point forecast	95% lower bound	95%‐upper bound
ARIMA‐TBATS	2022	9017.637	−667.306	18755.44
ARIMA‐TBATS	2023	9114.858	−9785.689	28262.70
ARIMA‐TBATS	2024	9181.405	−18896.782	37639.99
ARIMA‐TBATS	2025	9226.957	−27634.821	46560.23
ARIMA‐TBATS	2026	9258.137	−35865.759	54915.89
ARIMA‐TBATS	2027	9279.479	−43563.147	62698.65
ARIMA‐TBATS	2028	9294.088	−50748.882	69942.82
ARIMA‐TBATS	2029	9304.087	−57464.667	76698.60
ARIMA‐TBATS	2030	9310.932	−63758.110	83019.42

**Table 7:** Comparison of the best single and hybrid time series models.

Comparison of the best single and hybrid time series models					
Model	ME	RMSE	MAE	MPE	MAPE
TBATS	594.179	722.162	594.179	7.144	7.144
ARIMA‐TBATS	540.83	663.81	540.83	6.49	6.49

### 3.5 Mechanistic SIR Modeling Results

**Figure 8:**
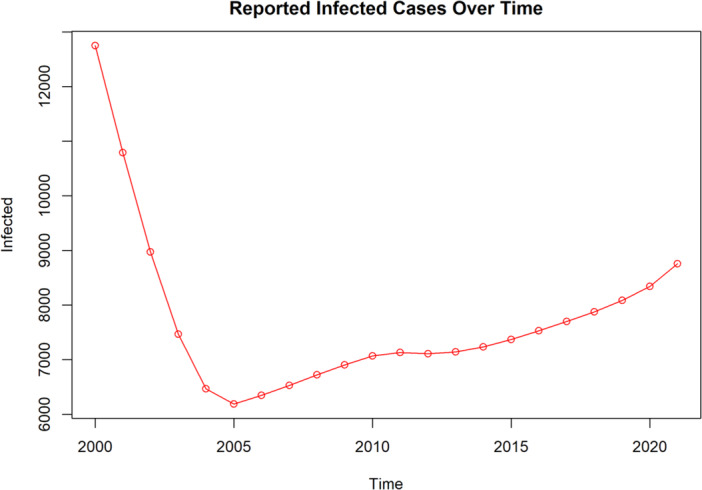
Reported infected cases over time.

The results of the SIR modeling analysis focused on the period of recent stabilization. Figure 8 illustrates the reported infected cases from the year 2000 to 2020, highlighting a gradual but steady increase in infections following the 2005 low point. The modeling utilized a starting population denominator (N_0_) of 8,721,465 individuals shown in Table 8. To account for population instability and growth over the multi‐decade study period, a net demographic birth/death rate (μ) was integrated directly into the SIR compartments. Rather than relying on external literature values, the transmission (β) and recovery (γ) parameters were estimated directly from the Somali historical dataset using a least‐squares optimization algorithm to minimize the sum of squared residuals between the model output and actual reported incidence. As listed in Table 9, the estimated annual transmission rate (β) was 46.5718, the recovery rate (γ) was 43.3224, and the net birth/death rate (μ) was 2.5517.

**Table 8:** Initial conditions of the model.

Compartment	Value
Susceptible (S_0_)	8,708,713
Infected (I_0_)	12,752
Recovered ($R_{0}$)	0
**Total Population (N_0_)**	**8,721,465**

**Table 9:** Best‐fit parameters for the sIR model.

Parameter	Symbol	Estimated Value (per year)
Transmission rate	*β* (beta)	46.5718
Recovery rate	*γ* (gamma)	43.3224
Net birth/death rate	*µ* (mu)	2.5517

**Figure 9:**
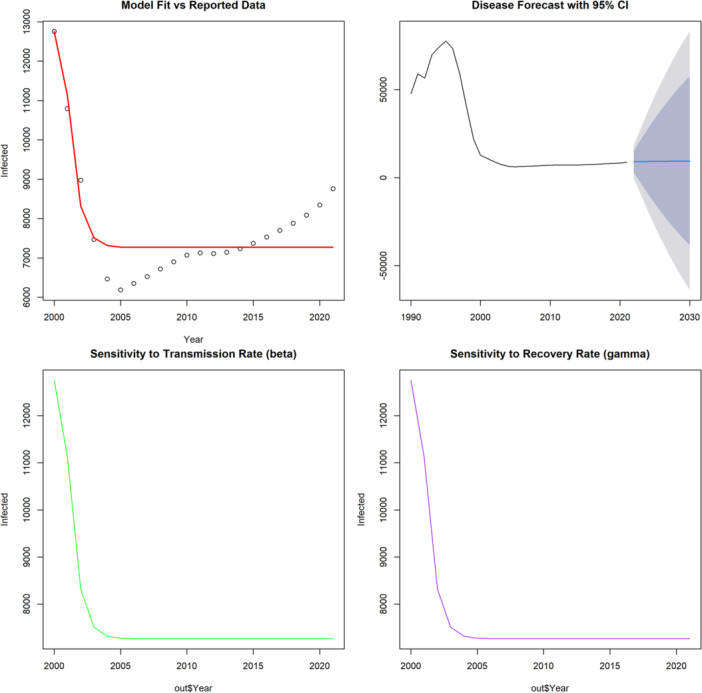
SIR Model results.

The performance of the fitted SIR model was evaluated by comparing its output against reported data, with the key metrics summarized in Table 10. The model achieved a MAPE of 6.75%, indicating a reasonably good fit for a mechanistic framework. Figure 9 provides a comprehensive 4‐panel analysis of the SIR results. The “Model Fit vs Reported Data” panel shows the SIR model (red line) smoothing out the annual fluctuations to capture the macro‐trend. The sensitivity analysis panels for *β* and γ demonstrate how variations in transmission and recovery rates would dramatically alter the epidemic's trajectory.

**Table 10:** Model fit evaluation metrics.

Metric	Value
Mean absolute error (MAE)	500.26
Root mean squared error (RMSE)	641.40
Mean absolute percentage error (MAPE)	6.75%

Finally, the forecasted incident for the next 9 years using the SIR model is presented in Table 11. These projections, combined with a calculated Basic Reproduction Number $R_{0}$ of 1.015, support the conclusion that the disease will persist and remain endemic within the Somali population, as shown by the stable median forecasts in Table 11.

**Table 11:** Forecasted incidence (next 9 years) with 95% CI.

Year	Lower 95% CI	Median Forecast	Upper 95% CI
2022	7266.43	7267.03	7267.58
2023	7266.63	7267.03	7267.44
2024	7266.69	7267.03	7267.35
2025	7266.71	7267.03	7267.33
2026	7266.72	7267.03	7267.33
2027	7266.72	7267.03	7267.33
2028	7266.72	7267.03	7267.33
2029	7266.72	7267.03	7267.33
2030	7266.72	7267.03	7267.33

## 4 Discussion

This study rigorously compared the performance of mechanistic, statistical, and hybrid models for forecasting dengue fever incidence in Somalia, a region critically vulnerable to climate change and characterized by a fragile health system. The findings reveal a compelling narrative: while individual time series models like TBATS offer a competent baseline for prediction, the synergistic power of hybrid approaches, specifically ARIMA‐TBATS, significantly enhances forecasting accuracy. The consistent outperformance of ARIMA‐TBATS across key error metrics—achieving a MAPE of 6.49% and an RMSE of 663.81 underscores the value of combining models to capture the multifaceted linear and non‐linear dynamics of dengue transmission in data‐scarce environments. As noted by Keeling & Rohani [1], the selection between mechanistic and statistical approaches is often a trade‐off between explaining the ‘why’ and predicting the ‘what’; however, our results suggest that hybridization bridges this gap by leveraging the strengths of both paradigms to overcome individual limitations [2].

The superior performance of the ARIMA‐TBATS model in the Somali context provides an interesting contrast to studies in other geographical regions. For instance, while Earnest et al. [4] found ARIMA models effective for dengue notifications in Singapore, and Akter et al. [5] identified neural networks as superior in Dhaka, Bangladesh, our study highlights that for the specific epidemiological landscape of the Horn of Africa, a hybrid approach is most robust. This aligns with recent local precedents in Somalia where ARIMA‐TBATS was found to outperform individual models in other complex time‐series tasks, such as forecasting CO_2_ emissions [16]. By utilizing an ensemble averaging method, the hybrid model effectively smoothed the volatility inherent in Somali public health data, which is often marked by under‐reporting and gaps [7]. This suggests that the “one‐model‐fits‐all” approach is insufficient for high‐stakes public health scenarios in resource‐constrained settings.

Furthermore, this hybrid framework demonstrates high generalizability to other conflict‐affected, data‐sparse settings in the Horn of Africa and the Middle East (such as Sudan or Yemen), where high‐resolution surveillance is often disrupted by instability. In these regions, this hybrid framework provides a highly resilient methodology for extracting stable macro‐trends from volatile, low‐quality aggregate data. Beyond pure forecasting, the mechanistic SIR model provided foundational insights into the long‐term disease dynamics within the population. The calculation of a basic reproduction number $R_{0}$ of 1.015 indicates that dengue fever has reached an endemic state in Somalia, as any value greater than 1 suggests the infection can sustain its spread [13]. The stability analysis further confirmed that the Disease‐Free Equilibrium (DFE) is unstable, meaning the disease will not naturally die out without targeted interventions. While the SIR model's practical application was challenged by the need for precise parameter estimation a difficulty previously noted in regions with inconsistent data collection [8] its ability to model population shifts between susceptible, infected, and recovered states offers a macro‐level understanding that complements the short‐to‐medium‐term actionable intelligence provided by the statistical models.

It is important to acknowledge that the SIR model, traditionally designed for high‐frequency outbreak tracking, serves here as a macro‐level diagnostic tool rather than a short‐term predictor. By applying it to annual aggregate data with vital dynamics, we successfully capture the long‐term endemic equilibrium of dengue in Somalia. This approach addresses the physical trajectory of the disease, allowing us to derive the basic reproduction number ($R_{0}=1.015$), which provides public health officials with a threshold‐based understanding of disease persistence that statistical models alone cannot provide.

Finally, the integration of both statistical and mechanistic approaches represents a significant advancement in addressing the unique epidemiological challenges faced by Somalia. The data‐driven models provide the precision necessary for immediate planning, while the SIR model offers a theoretical framework for understanding disease persistence. This dual approach is particularly crucial given Somalia's susceptibility to climate change, which continues to expand the habitat of the mosquito vector [6]. By establishing a scientifically validated baseline, this research fills a critical gap in the literature regarding dengue fever in the Horn of Africa, providing a methodological roadmap for other researchers working in similarly data‐scarce and high‐vulnerability environments [14].

### 4.1 Strengths and Limitations

A primary strength of this study is its status as the first comprehensive, side‐by‐side comparative analysis of 21 different modeling frameworks specifically tailored to the Somali context. By evaluating mechanistic, statistical, and hybrid models simultaneously, we have provided a robust evidence‐based infrastructure for the country's public health system. However, several limitations must be acknowledged. The analysis relied on annual incidence data, which, while useful for long‐term trends, may smooth out critical intra‐seasonal fluctuations critical for vector control timing (T [5]). Furthermore, the models were developed in a data‐scarce environment without real‐time climate covariates such as rainfall and temperature [9].

Furthermore, due to the extreme brevity of the dataset (*N* = 32), we utilized a single chronological train‐test split (1990–2016/2017–2021) rather than rolling‐origin cross‐validation. Cross‐validation would have resulted in training folds too small for complex algorithms like NNAR or TBATS to mathematically converge, though this limits our ability to report out‐of‐sample variability. The observed “widening” of confidence intervals in the long‐term forecasts (as seen in Figure 7) reflects the inherent uncertainty of projecting epidemiological trends years into the future without these dynamic inputs. While the current model is constrained by aggregate data lacking pediatric or demographic stratification, the mathematical architecture of the ARIMA‐TBATS ensemble is pathogen‐agnostic. This ensures that the framework can be rapidly redeployed to forecast other critical climate‐sensitive, vector‐borne diseases in the Horn of Africa, such as Malaria or Rift Valley Fever, providing a versatile tool for regional health ministries.

### 4.2 Policy Implications

The findings of this research have immediate practical applications for Somali health authorities and international partners. We strongly recommend the adoption of the ARIMA‐TBATS hybrid model as the core of a national Dengue Early Warning System (DEWS). Such a system would empower health officials to proactively allocate scarce resources, implement targeted vector control measures, and mobilize medical supplies before an outbreak reaches its peak. By shifting from a reactive to a proactive public health strategy, the implementation of these forecasting tools has the potential to save lives and reduce the burden on Somalia's fragile healthcare infrastructure [7].

While our model currently relies on aggregate national incidence data, it compensates for the lack of granular demographic data by utilizing a hybrid ensemble that treats the population as a single, interdependent unit. This pathogen‐agnostic structure, combined with the SIR‐derived endemicity threshold, ensures that the framework remains applicable even in the absence of stratified surveillance. By identifying the endemic baseline, health authorities can prioritize resources for universal interventions such as city‐wide fumigation and bed net distribution thereby shifting from reactive management to a proactive strategy despite data limitations.

### 4.3 Future Work

Future research should focus on refining these hybrid models by incorporating higher‐resolution temporal data, such as weekly or monthly notifications, to capture seasonal spikes more accurately. Additionally, the integration of real‐time environmental and climatic data specifically rainfall and precipitation patterns could significantly improve the predictive precision of the models [8]. Investigating the impact of socio‐economic factors on dengue transmission within Somalia would also provide valuable insights for more targeted interventions. Finally, developing user‐friendly interfaces for these complex models will be essential to ensure their accessibility for local public health stakeholders.

## 5 Conclusion

This study successfully established an optimal forecasting framework for dengue fever incidence in Somalia by comprehensively evaluating mechanistic, statistical, and hybrid modeling paradigms. In the absence of reliable historical climate covariates (such as rainfall and temperature data), the ARIMA‐TBATS hybrid model emerged as the most empirically effective univariate tool… This study provides a necessary foundation for future efforts to integrate real‐time climatic and environmental covariates once such surveillance infrastructure matures. This performance significantly surpassed the best single model (TBATS), underscoring the critical importance of synergistic modeling approaches in regions with complex epidemiological landscapes and data limitations. Furthermore, the SIR model validated the physical endemic nature of the disease (*R*_0_ = 1.015). Because the transmission rate is hovering just above the critical *R*_0_ = 1.0 replacement threshold, this physically implies that even modest, highly‐targeted vector control interventions could successfully break the chain of transmission and drive the pathogen toward local elimination. By providing the first scientifically validated forecasting instrument tailored to Somalia's challenging data environment, this research offers a vital piece of evidence‐based infrastructure for the country's public health system. These results provide a robust blueprint for proactive resource allocation and the development of early warning systems, representing a significant step forward in addressing vector‐borne diseases in resource‐restricted environments globally.

## Author Contributions

**Abdiftah Mohamud Abdi:** investigation. **Saralees Nadarajah:** investigation. **Abdisalam Hassan Muse:** investigation.

## Funding

The authors have nothing to report.

## Ethics Statement

Ethical approval and informed consent were not required for this study as it utilized exclusively aggregated, publicly available secondary data.

## Conflicts of Interest

The authors declare no conflict of interest.

## Transparency Statement

The corresponding author affirms that this manuscript is an honest, accurate, and transparent account of the study being reported; that no important aspects of the study have been omitted; and that any discrepancies from the study as planned have been explained.

## Data Availability

The data that support the findings of this study are openly available in https://microdata.nbs.gov.so/index.php/catalog/50 at https://microdata.nbs.gov.so/index.php/catalog/50.
